# Bonding Performance of Etch-and-Rinse and Universal Adhesives for Metal Bracket Fixation: An In Vitro Mechanical and SEM Study

**DOI:** 10.3390/biomedicines14051157

**Published:** 2026-05-20

**Authors:** Cristina Iosif, Anca Labunet, Andreea Kui, Stanca Cuc, Marioara Moldovan, Sorina Sava

**Affiliations:** 1 Dental Materials Discipline, Department 4—Prosthodontics and Dental Materials, Faculty of Dental Medicine, “Iuliu Hatieganu” University of Medicine and Pharmacy, 400012 Cluj-Napoca, Romania; mecicris@yahoo.com (C.I.); savasorina@elearn.umfcluj.ro (S.S.); 2 Prosthetic Dentistry Discipline, Department 4—Prosthodontics and Dental Materials, Faculty of Dental Medicine, “Iuliu Hatieganu” University of Medicine and Pharmacy, 400012 Cluj-Napoca, Romania; 3 Institutul de Chimie “Raluca Ripan”, Universitatea “Babes-Bolyai”, 400294 Cluj-Napoca, Romania

**Keywords:** orthodontic, adhesion, bracket, shear bond strength

## Abstract

**Background:** Durable adhesion between orthodontic brackets and enamel is essential for successful fixed orthodontic therapy. Despite simplified adhesive systems being available, conventional etch-and-rinse adhesives remain widely used due to their reliable enamel bonding. **Methods:** This in vitro study evaluated the bonding performance of three orthodontic adhesive strategies in combination with Transbond XT composite resin for metal bracket fixation. Thirty extracted human premolars were randomly allocated to three groups according to the adhesive system applied: OptiBond Solo Plus (etch-and-rinse method), SafeBond Universal DC (selective enamel etching method) and Transbond XT primer (control method). Shear adhesion resistance, maximum force and breakout force were measured and statistically analysed. **Results:** No statistically significant differences were observed between the OptiBond and SafeBond groups for any of the evaluated mechanical parameters (*p* > 0.05), although the OptiBond group exhibited higher mean values. The Transbond XT primer group showed significantly lower adhesion resistance and debonding forces than both of the other groups (*p* < 0.05). SafeBond demonstrated lower variability of results compared with OptiBond. **Conclusions:** When used with Transbond XT composite resin, both OptiBond Solo Plus and SafeBond Universal DC provided comparable mechanical performance for metal bracket bonding. While OptiBond yielded higher mean bond strength values, SafeBond exhibited more consistent behaviour. The Transbond XT primer alone resulted in inferior bonding performance.

## 1. Introduction

In fixed orthodontic therapy, maintaining the integrity of the adhesive bond between the brackets and the enamel is crucial to achieving successful treatment outcomes. The bonding interface must withstand masticatory forces, orthodontic loads and functional stress over extended periods while enabling safe bracket removal without causing permanent enamel damage. Recurrent bracket debonding can prolong treatment duration, increase clinical costs and reduce efficiency, whereas excessively strong adhesion can result in enamel fractures during debonding [1,2].

Adhesion in orthodontics is achieved through physical, chemical and mechanical interactions. A stable and durable interface must be established between the dental substrate and the bonding material to ensure adequate retention [3,4].

The bonding procedure typically begins with enamel surface preparation, involving cleaning, drying and controlled demineralisation using phosphoric acid. Etching with 35–50% phosphoric acid for 20–30 s removes the intercrystalline enamel layer and creates microporosities that enhance surface energy and allow resin monomers to penetrate the enamel structure [3,4,5].

Rinsing eliminates residual acid, calcium phosphate salts and demineralised enamel debris from the surface [6]. After drying, properly etched enamel has a uniform matte appearance, which indicates that the surface has been effectively conditioned. Inadequate etching or residual debris may compromise resin infiltration, requiring repetition of the conditioning procedure [6].

Following etching, a primer is applied to improve surface wettability and facilitate interaction between the conditioned enamel and the adhesive resin [2,7]. The adhesive component itself is a low-viscosity, hydrophobic resin composed of polymerizable monomers capable of penetrating the etched enamel microstructure [8,9]. Upon polymerization, the adhesive forms resin extensions that provide micromechanical retention and contribute to the durability of the bonded interface by limiting marginal leakage and nano-infiltration [10].

Advances in dental materials have led to the development of various adhesive strategies. Some systems combine acidic components with primers (self-etch systems); others integrate all components into a single solution. As the need for customized, biocompatible, and visually appealing dental solutions rises, leveraging advanced technologies and personalized care, adapted to the medical professional’s interest and capabilities presents exciting opportunities for innovation [11]. Conventional etch-and-rinse systems, meanwhile, maintain a separate etching step, followed by the application of a primer and adhesive [4]. Despite the availability of these simplified systems, however, the clinical success of fixed orthodontic appliances remains strongly dependent on the quality of the adhesive interfaces. Adhesion results from molecular interactions between the substrate and the adhesive when intimate and uniform contact is achieved across the bonding surface [3,4,12].

In orthodontic bonding, the two substrates involved are the enamel surface and the bracket base, with the adhesive layer mediating force transfer between them. The continuous development of new materials and modifications to existing systems means that clinicians must understand the properties, composition and limitations of adhesive cements, as well as the factors that influence their clinical performance [13,14].

The most commonly used orthodontic bonding agents are composite resin-based cements, primarily Bis-GMA-based materials, alongside resin-modified glass ionomers [15]. The mechanical properties of resin cements depend on filler content, filler–matrix coupling, and resistance to water absorption [7]. Polymerization shrinkage and water sorption can influence marginal integrity, chromatic stability and long-term performance [16,17]. Transbond XT is consistently regarded as a gold-standard orthodontic adhesive due to its reliable and clinically acceptable shear bond strength. Evidence from the reviewed studies shows that it consistently achieves bond strength values above the minimum threshold required for successful bracket adhesion, ensuring low failure rates and stable performance. Its mechanical properties provide effective retention of metallic brackets while maintaining a favorable balance between strong adhesion and safe debonding, making it a widely accepted reference material in orthodontic bonding research [18,19].

Metal brackets remain the most frequently used orthodontic appliance and are typically fabricated from stainless steel or other metallic alloys. Their retention relies exclusively on mechanical interlocking between the bracket base and the adhesive layer, as chemical bonding to enamel is not possible. Consequently, extensive research has focused on optimizing bracket base design to enhance micromechanical retention, as the size and configuration of the bracket base significantly influence bond strength [9].

During bracket debonding, failure may occur at the bracket–adhesive interface, the adhesive–enamel interface, or within the adhesive layer itself. Greater adhesion to enamel increases the risk of enamel damage, so controlled failure at the bracket–adhesive interface is generally favored [5]. Bracket detachment rates reported in the literature range from 0.5% to 17.6%, depending on clinical technique, enamel characteristics, contamination, patient-related factors and mechanical loading conditions [20,21].

Despite significant progress in adhesive technology, two-step etch-and-rinse systems remain the clinical reference standard for enamel bonding thanks to their reliable micromechanical interaction and consistent performance [22]. However, the growing use of universal adhesives in clinical practice, including dual-cure formulations, requires further evaluation of their suitability for orthodontic applications. The performance of universal adhesives when applied to dental hard tissues and indirect restorative materials is material-dependent, as not all formulations are recommended for bonding to every type of restorative substrate. With regard to adhesion to dental tissues, selective enamel etching with phosphoric acid prior to the application of universal adhesives has been shown to enhance bond strength and improve marginal sealing [23].

With this in mind, the present study aimed to compare the influence of two different adhesive systems on the strength of the adhesive bond between orthodontic metal brackets and enamel, using a widely accepted orthodontic bonding system as a reference. Specifically, metal brackets bonded using Transbond XT composite resin and primer—considered the clinical gold standard for orthodontic bonding [24,25]—were compared with two alternative adhesive strategies: a two-step etch-and-rinse adhesive system (OptiBond Solo Plus) and a dual-cure universal adhesive applied using a selective etching protocol (SafeBond).

The primary research question was whether using an etch-and-rinse adhesive system results in differences in bond strength between orthodontic brackets and enamel when a composite-based cement is used, compared with using a selectively etched dual-cure universal adhesive. The secondary research question addressed potential differences in the amount and surface characteristics of residual adhesive material on enamel and bracket surfaces after debonding, as evaluated by scanning electron microscopy.

Although orthodontic bracket bonding has been extensively studied, ongoing developments in adhesive formulations and inconsistencies in reported bond strength values and failure modes highlight the need for standardized investigations combining mechanical testing with microstructural analysis. This study aims to correlate quantitative adhesion data with a morphological evaluation of debonding behavior to provide clinically relevant information regarding bond strength, failure patterns and enamel preservation.

## 2. Materials and Methods

### 2.1. Ethical Approval and Study Design

This in vitro experimental study was conducted with the approval of the Ethics Committee of the “Iuliu Hațieganu” University of Medicine and Pharmacy, Cluj-Napoca (approval no. 133, 27 June 2023). All experimental procedures were carried out at the Dental Materials and Ergonomics Discipline of the same institution, in collaboration with the ‘Raluca Ripan’ Institute of Chemistry in Cluj-Napoca.

### 2.2. Tooth Selection and Preparation

Thirty human maxillary and mandibular premolars, extracted for orthodontic or periodontal reasons, were selected. The inclusion criteria were the absence of carious lesions, restorations, structural defects or enamel cracks, whether congenital or acquired. Teeth with visible damage or previous chemical treatment were excluded.

Following extraction, any remaining soft tissue and deposits were removed using an ultrasonic scaler. The teeth were then professionally brushed with a fluoride-free prophylactic paste (Lunos^®^ Prophy Paste, Dürr Dental, SE, Bietigheim-Bissingen, Baden-Württemberg, Germany). The specimens were then stored in distilled water at room temperature until use. Brackets were bonded to the vestibular surface of all premolars.

### 2.3. Group Allocation and Materials

After cleaning, the teeth were randomly allocated to three experimental groups (n = 10) according to the adhesive system used for bracket bonding. The same resin composite cement (Transbond XT, 3M, Unitek, Monrovia, CA, USA) was used for bracket fixation in all groups.

The adhesive systems and application strategies for each group are summarized in Table 1.

Group 1 (G1): OptiBond Solo Plus (Kerr), a two-step etch-and-rinse adhesive.

Group 2 (G2): SafeBond Universal DC (Schulzer)—dual-cure universal adhesive applied with selective enamel etching.

Group 3 (G3, control): Transbond XT Adhesive Primer (3M), a two-step etch-and-rinse system.

Metal orthodontic brackets (0.022-inch slot, stainless steel, Roth prescription, Orthofocus Visio 2.0) were used in all groups. Light curing was performed using an O-Star ‘One Cure’ LED curing unit (Woodpecker, Guilin Woodpecker Medical Instrument Co., Ltd., Guilin, China) with a wavelength range of 385–515 nm and a light intensity of 2700–3000 mW/cm^2^.

### 2.4. Adhesive Application Protocols

#### 2.4.1. Etch-and-Rinse Adhesive (Group 1)

For Group 1 specimens, the enamel surfaces were etched with 36% orthophosphoric acid gel (Blue Etch, Cerkamed Medical Company, Stalowa Wola, Poland) for 30 s. The surfaces were then rinsed thoroughly with water and air-dried for 2–3 s.

OptiBond Solo Plus adhesive was then applied according to the manufacturer’s instructions. Two consecutive layers were brushed onto the enamel surface for 15 s each, with gentle air drying for three seconds after each application. The adhesive was then light-cured for 20 s.

#### 2.4.2. Dual-Cure Universal Adhesive with Selective Etching (Group 2)

In Group 2, selective enamel etching was performed using 36% orthophosphoric acid, which was applied to the enamel surface for 15 s. This was followed by rinsing and drying.

SafeBond Universal DC adhesive was prepared by mixing one drop each from bottles A and B for 10 s. Two layers were applied to the enamel surface using continuous brushing for 30 s per layer, followed by air drying for 10 s. The adhesive was then light-cured for 20 s.

#### 2.4.3. Transbond XT Adhesive Primer (Control Group 3)

For the control group, the enamel surfaces were etched with 36% orthophosphoric acid for 30 s, then rinsed and dried. Transbond XT primer was applied by brushing for 10 s, followed by gentle air drying for 2 s. No light curing was performed, as polymerisation occurs during composite curing.

The adhesive system used in each group, as well as the adhesion strategy, is shown in Table 2.

### 2.5. Bracket Bonding Procedure

In all groups, Transbond XT composite resin was applied in a thin layer to the bracket base. The brackets were positioned on the mid-buccal surface of each tooth. Excess resin was then removed, and the brackets were light-cured for 20 s.

Each tooth was then embedded vertically in self-curing acrylic resin (Duracryl Plus, Spofa Dental, under Kerr Corporation, Jičín, Hradec Králové, Czech Republic), ensuring that the crown was exposed up to 2 mm below the cemento-enamel junction to standardise positioning for mechanical testing.

### 2.6. Shear Bond Strength Testing

**Figure 1:**
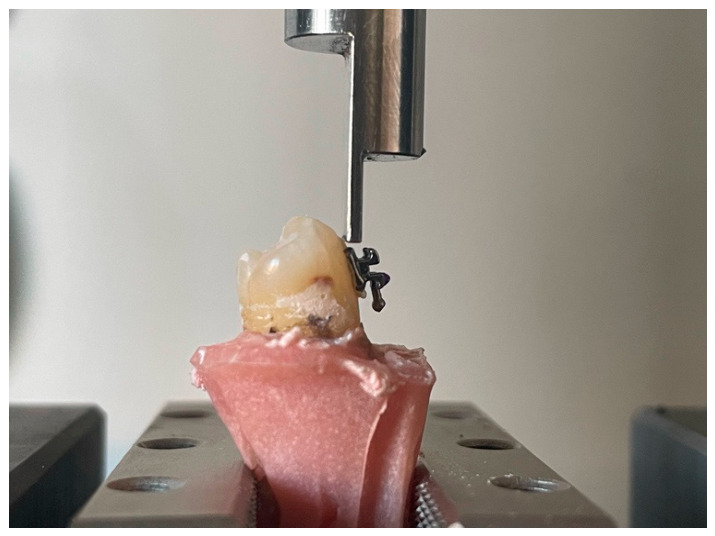
Shear adhesion test.

Shear adhesion resistance was measured using a universal testing machine (Lloyd LR5k Plus, Ametek/Lloyd Instruments, AMETEK Lloyd Instruments, Fareham, UK) with a load capacity of 5 kN. A shear force was applied in the occlusal-gingival direction at a crosshead speed of 1 mm/min, with a load of 0.5 N (Figure 1).

Force values were recorded in Newtons (N) and converted to megapascals (MPa). Data acquisition and processing were performed using NEXYGENPlus Version 3.0.

### 2.7. Statistical Analysis

For each group, shear adhesion resistance, maximum force and breakout force were calculated as the mean ± standard deviation. Statistical analysis was performed using Origin 2019b Graphing & Analysis software, OriginLab Corporation, Northampton, MA, USA.

Normality was assessed prior to analysis. Comparisons between groups were conducted using one-way analysis of variance (ANOVA), with a significance level of α = 0.05. Post hoc comparisons were performed when appropriate.

### 2.8. Scanning Electron Microscopy (SEM)

After debonding, two specimens were selected from each group for morphological evaluation. The enamel surfaces and bracket bases were examined using a scanning electron microscope (Inspect™ S, FEI, Hillsboro, OR, USA).

Observations were conducted under low vacuum at accelerating voltages of 4.0 kV and 20 kV, and at magnifications of 50×, 100×, 500× and 1000×. The SEM analysis focused on characterizing the failure mode, the distribution of residual adhesive, and the surface morphology of the enamel and bracket bases following debonding.

## 3. Results

### 3.1. Shear Adhesion Resistance and Debonding Forces

**Figure 2:**
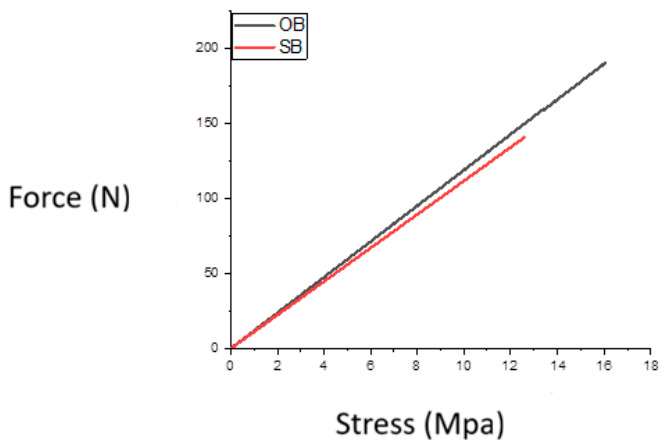
Adhesion test curve for OptiBond (OB) and SafeBond (SB) adhesive.

Figure 2 shows representative load–displacement curves obtained during shear testing for Groups 1 and 2. In both groups, the curves exhibited a roughly linear relationship between the applied shear force and the recorded stress up to the point of failure. This was followed by a sudden drop, which corresponded to bracket detachment and was consistent with brittle failure behaviour.

The mean values and standard deviations for adhesion resistance, maximum force and breakout force for each group are presented in Table 3, Table 4 and Table 5.

OptiBond Solo Plus (G1) exhibited the highest mean values for all measured parameters, followed by SafeBond Universal DC (G2); meanwhile, the Transbond XT primer group (G3) exhibited the lowest values. There was variability among groups, with larger standard deviations observed in G1 than in G2 and G3.

One-way analysis of variance revealed statistically significant differences among the three adhesive systems for adhesion resistance (F(2, 27) = 8.52, *p* = 0.003), maximum force (F(2, 27) = 4.78, *p* = 0.025) and breakout force (F(2, 27) = 4.31, *p* = 0.033). Post hoc comparisons showed that G3 had significantly lower values for all parameters compared with G1 and G2 (*p* < 0.05). No statistically significant differences were observed between G1 and G2 for adhesion resistance, maximum force or breakout force (*p* > 0.05).

Direct comparison of the two experimental adhesive systems showed no statistically significant differences between OptiBond (G1) and SafeBond (G2) for adhesion resistance (F(1, 18) = 3.46, *p* = 0.092), maximum force (F(1, 18) = 1.52, *p* = 0.246) or breakout force (F(1, 18) = 1.25, *p* = 0.289).

**Figure 3:**
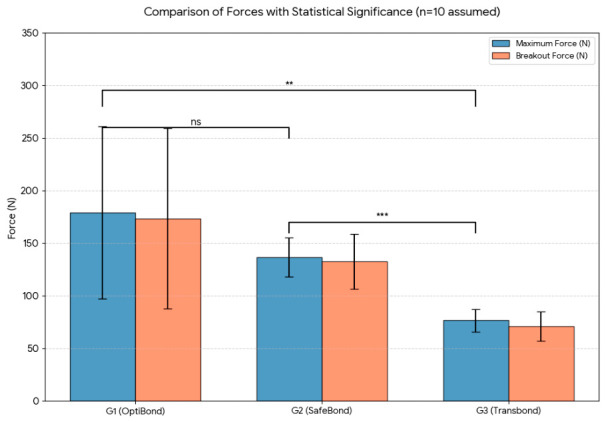
Comparison of forces with statistical significance n = 10, ns = not significant, **/*** = statistically significant.

A graphical comparison of adhesion resistance values among the three groups is shown in Figure 3.

### 3.2. Macroscopic Evaluation After Debonding

**Figure 4:**
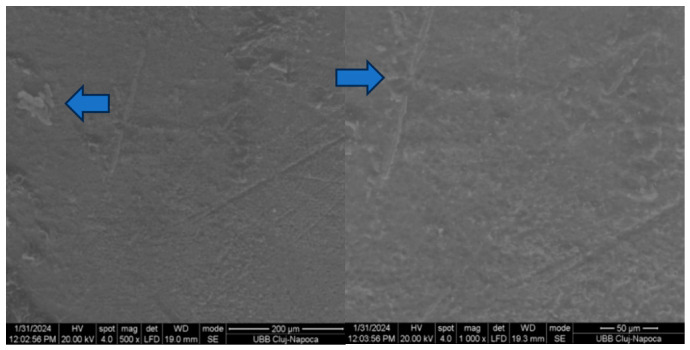
SEM appearance of the enamel of a Group 1 (OptiBond) tooth after detachment at 500× and 1000×. Arrows show adhesive on the surface.

**Figure 5:**
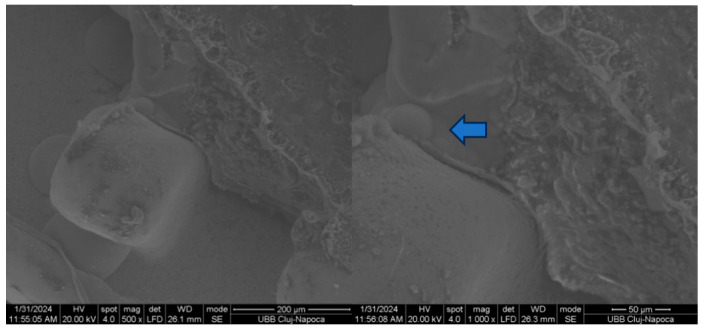
SEM aspect of the bracket of a group 1 tooth (OptiBond) after detachment at 500× and 1000×. Arrows show adhesive on the surface.

Figure 4 and Figure 5 illustrate the macroscopic examination of the enamel and bracket surfaces following debonding. Group 1 showed a higher proportion of residual adhesive material on the enamel surface after bracket removal, while Group 2 showed a higher amount of adhesive residue on the bracket base. No visible enamel fractures or cracks were observed in either group.

Based on the Adhesive Remnant Index (ARI), Group 1 predominantly demonstrated higher ARI scores of 2–3, indicating that more than 50% to nearly all of the adhesive remained on the enamel surface after debonding. In contrast, Group 2 showed lower ARI scores of 0–1, reflecting minimal adhesive remaining on the enamel surface and greater adhesive retention on the bracket base. This suggests that debonding was clinically safe, with failure occurring within the adhesive layer or at the adhesive interfaces rather than damaging enamel. ARI scoring considered 0 = no adhesive left on enamel, 1 = less than 50% adhesive left on enamel, 2 = more than 50% adhesive left on enamel, 3 = all adhesive left on enamel, with impression of bracket mesh [15].

### 3.3. Scanning Electron Microscopy (SEM) Analysis

**Figure 6:**
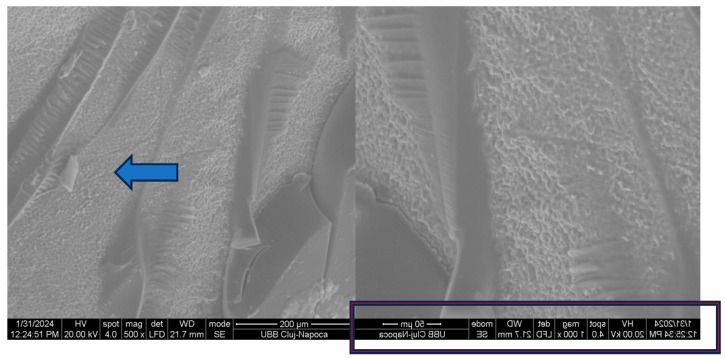
SEM appearance of the enamel of a group 2 (SafeBond) tooth after detachment at 500× and 1000× magnification. Arrows show bracket base-shaped adhesive on the surface.

**Figure 7:**
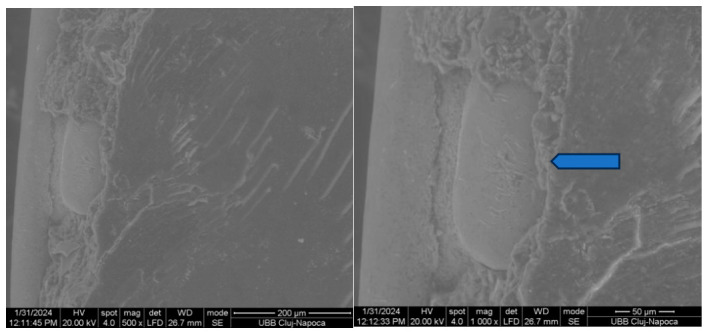
The SEM aspect of the bracket of a group 2 tooth (SafeBond) after detachment at 500× and 1000×. Arrows show bracket base-shaped adhesive on the surface.

Representative scanning electron microscope (SEM) images of enamel surfaces and bracket bases following debonding are shown in Figure 4, Figure 5, Figure 6 and Figure 7. SEM analysis was performed at magnifications ranging from 50× to 1000× on two specimens per group.

In both experimental groups, shear loading resulted in heterogeneous surface morphologies characterised by areas of exposed enamel interspersed with residual adhesive. The enamel surfaces exhibited localised irregularities corresponding to adhesive remnants, with no evidence of enamel prism fracture or structural discontinuity.

Examination of the bracket-adhesive interface showed that the retentive structures of the metal bases successfully retained adhesive material, confirming the efficacy of the mechanical interlock. These patterns of adhesive failure were heterogeneous across the enamel and bracket surfaces, in alignment with macroscopic assessments.

### 3.4. Stereomicroscopic Observations

Post-debonding stereomicroscopic analysis revealed heterogeneous distributions of residual adhesive across the enamel surfaces. Macroscopic evaluation indicated that the enamel remained structurally intact, with no evidence of iatrogenic cracking or delamination. Minor surface irregularities were localized to regions of adhesive or cohesive failure within the interfacial complex. These findings suggest that enamel integrity is preserved across all investigated adhesive systems during the bracket removal process.

Supplementary Materials, showing additional SEM images, are available for download.

## 4. Discussion

Although the bonding of orthodontic brackets has been broadly investigated, ongoing advancements in adhesive chemistry, particularly the growing clinical accessibility of universal and dual-cure adhesive systems, warrant further evaluation of their mechanical behaviour and debonding characteristics. This study addressed this need by combining quantitative shear adhesion testing with microstructural surface analysis, enabling correlation between bond strength values, failure patterns, and enamel preservation.

The selection of the adhesive systems evaluated in the present study was based on their distinct bonding strategies, chemical composition, and clinical application protocols, which represent two different contemporary approaches to orthodontic bracket bonding. Conventional etch-and-rinse adhesives remain widely regarded as the clinical gold standard for enamel bonding due to their well-documented ability to produce reliable micromechanical retention through phosphoric acid etching and resin tag formation. In contrast, universal adhesives have been developed to simplify adhesive procedures and increase clinical versatility by allowing their application in multiple modes, including self-etch, selective-etch, and etch-and-rinse strategies. Additionally, some universal systems incorporate dual-curing capabilities, enabling both light-activated and chemically initiated polymerization, which may enhance polymerization efficiency under orthodontic brackets where light penetration can be partially limited.

The comparison between these systems was therefore designed to evaluate whether newer universal adhesive technologies can achieve bonding performance comparable to conventional etch-and-rinse protocols in orthodontic applications. These materials differ not only in their etching strategy but also in the presence of functional monomers (e.g., 10-MDP), solvent composition, acidity, and polymerization mechanisms, all of which may influence enamel interaction, resin infiltration, and ultimately bond strength and failure patterns. Investigating these differences is clinically relevant because orthodontic bonding requires an optimal balance between sufficient bond strength to prevent bracket failure during treatment and controlled debonding at the end of therapy to minimize enamel damage. Therefore, comparing these adhesive strategies provides insight into whether simplified universal systems can serve as reliable alternatives to traditional protocols while potentially reducing technique sensitivity and chair time.

Several previous studies have compared different orthodontic bonding systems with Transbond XT, which is widely regarded as a clinical reference material [24,25,26,27,28,29,30]. A novel aspect of the present study is the evaluation of a dual-cure universal adhesive (SafeBond Universal DC), which was used in combination with a light-cured composite resin for metal bracket bonding. Dual-cure adhesive systems combine light-activated and chemical polymerization, which could offer advantages in orthodontic applications, especially under metal brackets where light transmission is restricted. This characteristic has the potential to improve polymerization completeness and bonding reliability.

The results of the present study demonstrated that the Transbond XT primer group exhibited significantly lower adhesion resistance, maximum force and breakout force than both the OptiBond Solo Plus and SafeBond Universal DC groups. In contrast, no statistically significant differences were observed between the two experimental adhesive systems. OptiBond Solo Plus showed higher mean values for all measured parameters, whereas SafeBond Universal DC demonstrated lower variability, as reflected by smaller standard deviations.

The mean adhesion resistance values obtained for OptiBond (16.97 MPa) and SafeBond (12.12 MPa) exceeded the minimum clinically acceptable bond strength range of 5.9–7.9 MPa proposed by Reynolds for orthodontic applications [31]. While OptiBond yielded higher mean values, the absence of statistically significant differences between the two groups suggests that SafeBond demonstrates comparable mechanical performance when applied using a selective enamel etching strategy.

The adhesion values obtained for the etch-and-rinse system are consistent with those reported by Hellak et al., who recorded mean shear bond strength values of approximately 15.5 MPa for Transbond XT when used with a conventional etching protocol [20]. In an in vitro study, self-etching adhesives demonstrated comparable bonding performance to total-etch systems. Similar findings have been reported in other investigations, which observed no significant differences in bracket debonding rates between self-etch primers and conventional etch-and-rinse systems while noting reduced clinical bonding time for self-etch approaches [28].

The literature presents heterogeneous findings regarding the performance of self-etch and universal adhesives. Some systematic reviews have reported a higher risk of bracket failure associated with self-etch primers over extended clinical observation periods, while also highlighting reduced bonding time as a practical advantage [26,30]. Other reviews have found no significant differences in failure rates between self-etch and acid-etch techniques at follow-up periods of up to 18 months [26]. The variability among vivo outcomes may be explained by differences in oral conditions, moisture control, patient-related factors and mechanical loading, which cannot be fully reproduced in vitro.

In the present study, SafeBond Universal DC demonstrated adhesion resistance values that are considered acceptable for clinical orthodontic bonding. This is in line with reports evaluating universal adhesive systems applied to enamel [32,33]. The use of selective enamel etching prior to the application of the universal adhesive contributed to the observed bond strength. This was achieved by enhancing micromechanical retention, while still preserving the simplified application protocol.

Several authors have reported that, under dry enamel conditions, conventional etch-and-rinse systems may yield higher bond strength values, whereas self-etch or universal adhesives may perform better in moist environments [34]. The dual-cure capability of SafeBond Universal DC is an added advantage as it allows for chemical polymerization to compensate for reduced light penetration beneath metal brackets. This contributes to more uniform curing of the adhesive layer. Although dual-cure composite materials have been studied extensively, data regarding dual-cure adhesive systems in orthodontics remain limited.

Failure during bracket debonding may occur at the bracket–adhesive interface, the adhesive–enamel interface or within the adhesive layer itself [35]. Excessively strong adhesion to enamel increases the risk of enamel damage during debonding, whereas insufficient adhesion can result in bracket failure during treatment. In the present study, scanning electron microscopy (SEM) analysis indicated effective mechanical retention at the bracket base and predominant failure at the adhesive–enamel interface, with no evidence of enamel prism fracture or structural damage.

The SEM findings confirmed the results of the mechanical tests and macroscopic observations. Residual adhesive was present on both the enamel and the bracket surfaces, and there were differences in its distribution between the two experimental adhesive systems. Enamel surfaces appeared intact, suggesting that adequate bond strength can be achieved without compromising enamel integrity when appropriate adhesive protocols are employed.

Several limitations of this study should be acknowledged. The sample size was small, and the in vitro design could not replicate all the complexities of the oral environment, such as thermal cycling, saliva, biofilm formation, and cyclic loading. Furthermore, the teeth were stored in distilled water rather than artificial saliva, which could have affected the properties of the enamel surfaces. These factors should be considered when extrapolating the results to clinical practice.

## 5. Conclusions

Within the limitations of this in vitro study, it can be concluded that the two experimental adhesive systems, OptiBond Solo Plus and SafeBond Universal DC, demonstrate comparable mechanical performance regarding shear adhesion resistance, maximum force, and breakout force when paired with Transbond XT composite resin. Both systems outperformed the Transbond XT primer control group, which exhibited significantly lower adhesion and debonding values. Notably, a trade-off between absolute strength and reliability was observed: OptiBond Solo Plus yielded higher mean bond strength values but was characterized by greater result variability, whereas SafeBond Universal DC provided a lower dispersion of values, suggesting more consistent mechanical behavior.

Furthermore, macro- and microscopic evaluations confirmed that enamel integrity was preserved across all tested groups, as evidenced by the absence of iatrogenic fractures or structural damage following bracket removal. These findings indicate that using a selectively etched dual-cure universal adhesive may represent a viable alternative, pending clinical validation, to conventional etch-and-rinse systems, maintaining mechanical efficacy while offering a more efficient clinical protocol. However, these results necessitate further validation through studies incorporating larger sample sizes and conditions that more closely simulate the complex oral environment to evaluate long-term clinical success.

## Figures and Tables

**Table 1:** Adhesive systems and application.

	Adhesive Systems	
Group Number (*n* = 10)	Commercial Product Name	Application Strategy
G1	OptiBond Solo Plus (Kerr Corporation, Brea, CA, USA)	Acid etching and rinsing, 2-step
G2	SafeBond Universal DC (Schulzer, P.L. Superior Dental Materials GmbH, Hamburg, Germany)	Self-etching, 1 dual time + selective etching
G3 Control	Transbond XT (3M, Unitek, Monrovia, CA, USA)	Acid etching and rinsing, 2-step

**Table 2:** Composition of adhesive systems.

Application and Adhesive Type	Trade Name/ Company	Composition	Type of Reaction
Etching & rinsing (2-step) Fifth Generation	OptiBond Solo Plus/Kerr	Bisphenol A Diglycidyl Ether Dimethacrylate (Bis-GMA), 2-hydroxyethyl methacrylate (HEMA), glicidil dimetacrilat, fluoro-silicates, silicium oxydes microfilled particles 0.4 μm, 15%	Light Curing
Self-Engraving (1 Beat) Generation VIII	SafeBond Universal DC/Schulzer	Bis-GMA, HEMA, Nanoparticles N/A	Dual (self-curing and light-curing)
Etching & rinsing (2-step) Fifth Generation	Transbond XT Adhesive Primer/3M	BISGMA, Triethylene Glycol Dimethacrylate (TEGDMA), initiators and stabilizers, no filling	No light curing is required, as the adhesive is embedded in the adhesive.

**Table 3:** Results of the G1 adhesion test (OptiBond).

G1 (OptiBond)	Adhesion Resistance (MPa)	Maximum Force (N)	Breakout Force (N)
**Mean**	16.97039	178.91664	173.31758
**Standard Deviation**	6.25482	81.94941	85.59494

**Table 4:** Results of the G2 adhesion test (SafeBond).

G2 (SafeBond)	Adhesion Resistance (MPa)	Maximum Force (N)	Breakout Force (N)
**Mean**	12.12177	136.65791	132.41854
**Standard Deviation**	1.26168	18.31444	26.16664

**Table 5:** Results of the G3 adhesion test (Transbond XT).

G3 (Transbond)	Adhesion Resistance (MPa)	Maximum Force (N)	Breakout Force (N)
**Mean**	6.46137988	76.76119298	70.90586222
**Standard Deviation**	1.009593251	10.72773095	13.93267591

## Data Availability

The data presented in this study are available on request from the corresponding author due to ethical reasons.

## Supplementary Materials

The following supporting information can be downloaded at https://www.mdpi.com/article/10.3390/biomedicines14051157/s1: SupplementarySEMImages.pdf. Supplementary figures: Figure S1. SEM appearance of the enamel of a Group 1 (OptiBond) tooth after detachment at (a) 50×, (b) 100×, (c) 500× and (d) 1000×; Figure S2. SEM aspect of the bracket of a Group 1 tooth (OptiBond) after detachment at (a) 100×, (b) 500× and (c) 1000×; Figure S3. SEM appearance of the enamel of a group 2 (SafeBond) tooth after detachment at (a) 50×, (b) 100×, (c) 500× and (d) 1000× magnification; Figure S4. The SEM aspect of the bracket of a group 2 tooth (SafeBond) after detachment at (a) 50×, (b) 100×, (c) 500× and (d) 1000×; Figure S5. SEM aspect of the bracket of a group 3 tooth (Transbond XT) after detachment at (a) 100×, (b) 450× and (c) 900× and (d) 1800×.

## Abbreviations

The following abbreviations are used in this manuscript:

ANOVA	Analysis of variance
ARI	Adhesive Remnant Index
Bis-GMA	Bisphenol A diglycidyl ether dimethacrylate
Bis-EMA	Bisphenol A bis(2-hydroxyethyl ether) dimethacrylate
MPa	Megapascal
N	Newton
SEM	Scanning electron microscopy
TEGDMA	Triethylene glycol dimethacrylate
