# Supplementary material for: Bonding Performance of Etch-and-Rinse and Universal Adhesives for Metal Bracket Fixation: An In Vitro Mechanical and SEM Study
Source: Biomedicines. 2026 May 20;14(5):1157. doi: 10.3390/biomedicines14051157 (PMC13204891; doi:10.3390/biomedicines14051157)
Supplement: Supplementary file 1 [file biomedicines-14-01157-s001.zip › biomedicines-4186067-supplementary.pdf]

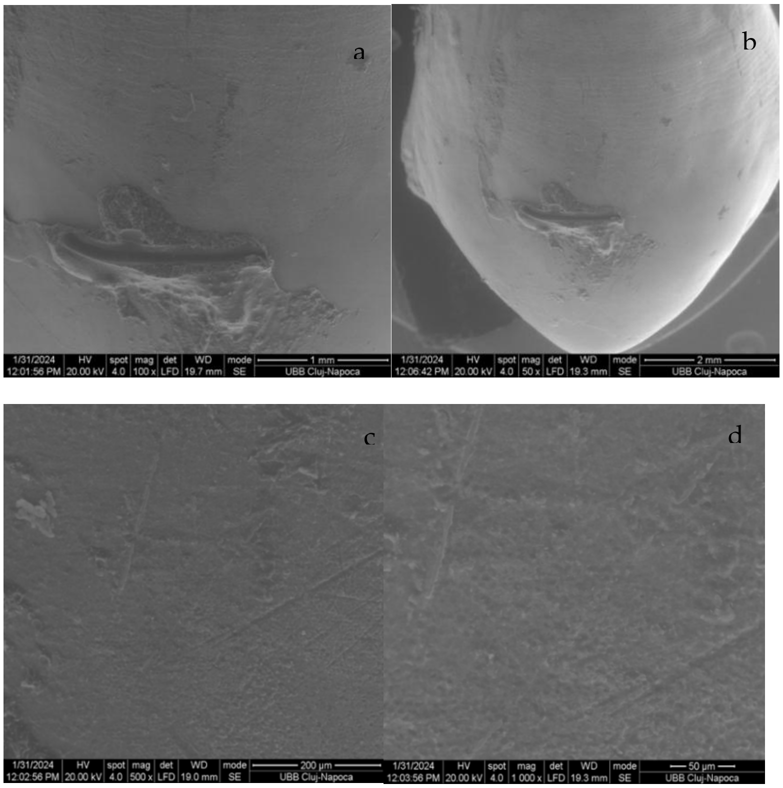

Figure S1. SEM appearance of the enamel of a Group 1 (OptiBond) tooth after detachment at a) 50x, b) 100x, c) 500x and d) 1000x.

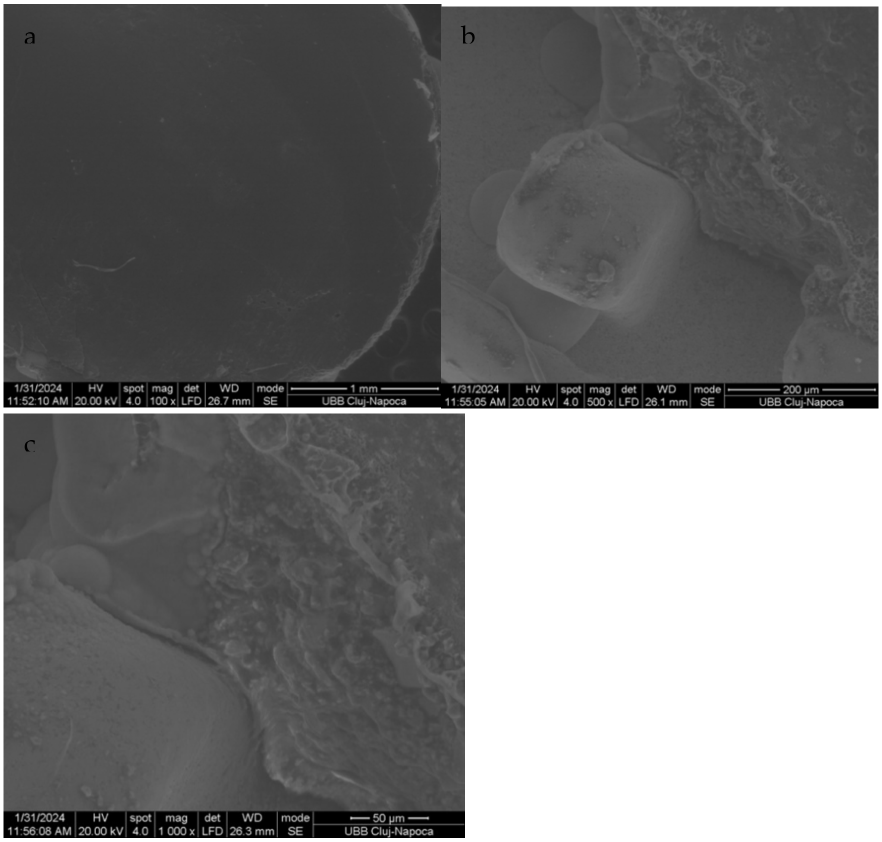

Figure S2. SEM aspect of the bracket of a Group 1 tooth (OptiBond) after detachment at a) 100x, b) 500x and c) 1000x.

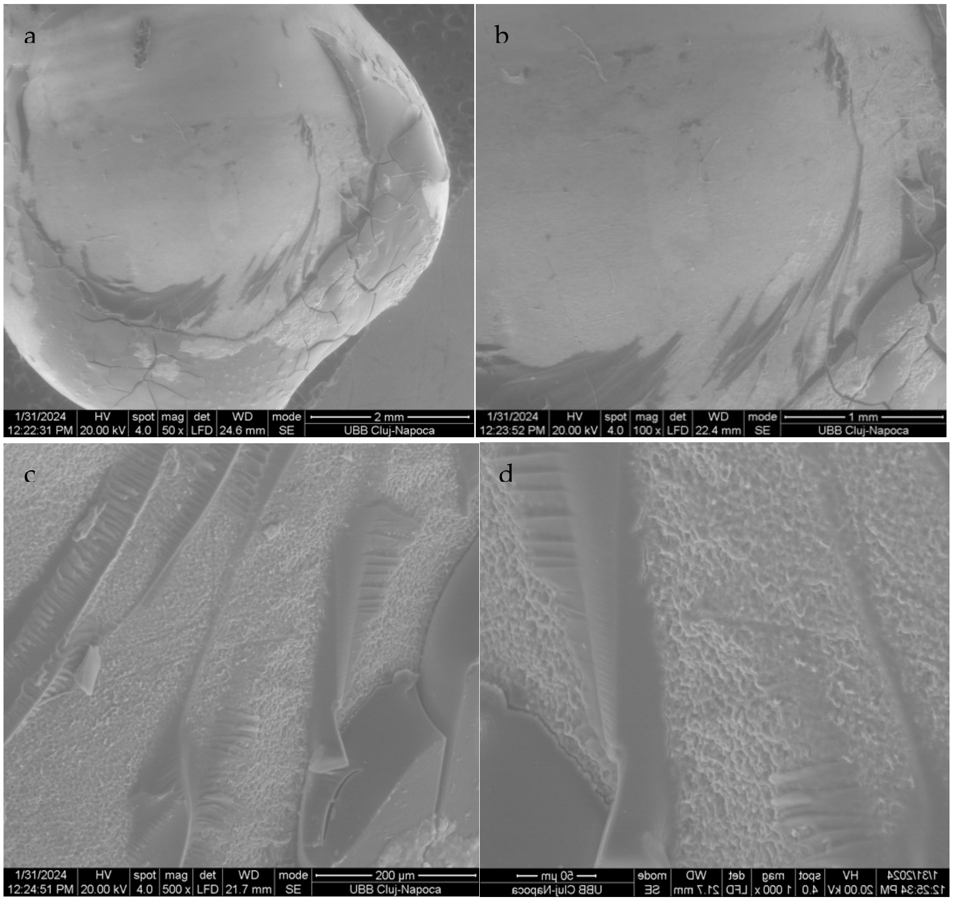

Figure S3. SEM appearance of the enamel of a group 2 (SafeBond) tooth after detachment at a) 50x, b) 100x, c) 500x and d) 1000x magnification.

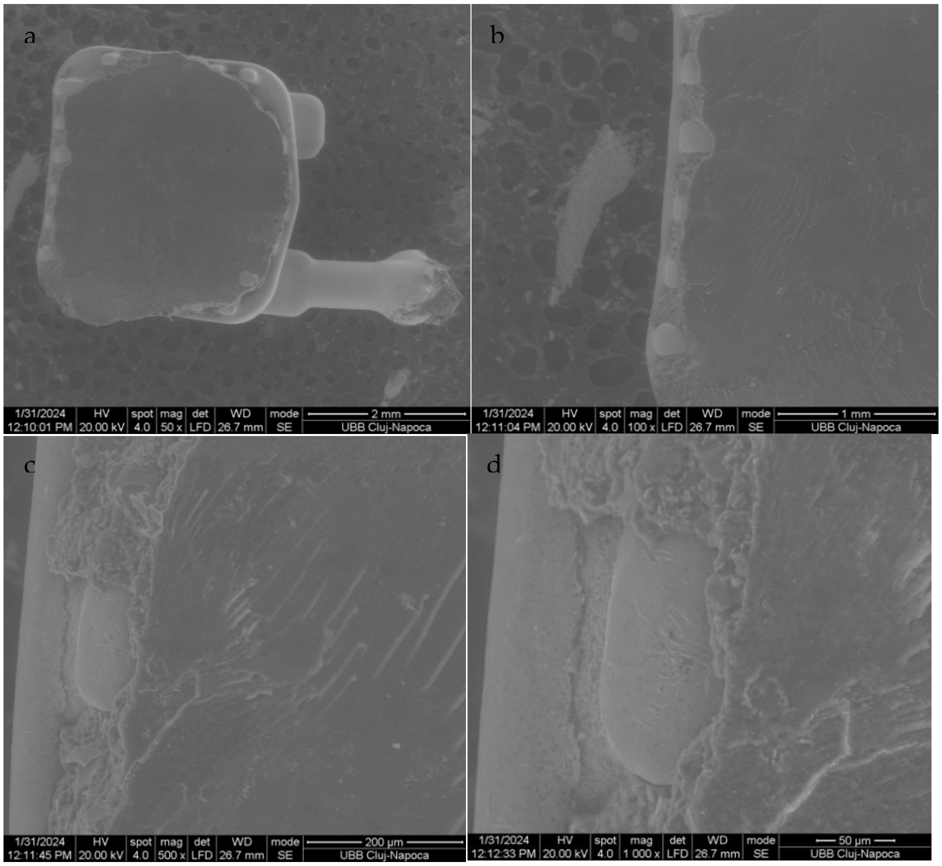

Figure S4. The SEM aspect of the bracket of a group 2 tooth (SafeBond) after detachment at a) 50x, b) 100x, c) 500x and d) 1000x.

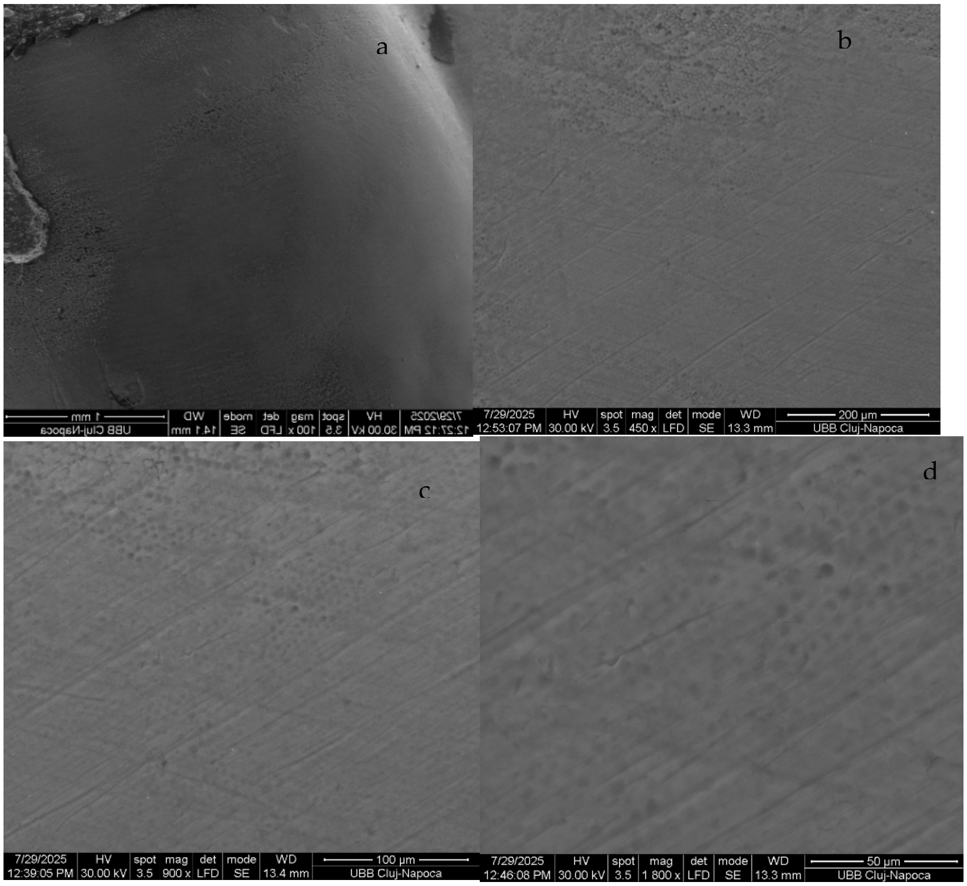

Figure S5. SEM aspect of the bracket of a group 3 tooth (Transbond XT) after detachment at a) 100x, b) 450x and c) 900x and d) 1800x.
